# Spatiotemporal Distribution of Homogalacturonans and Hemicelluloses in the Placentas, Ovules and Female Gametophytes of *Utricularia nelumbifolia* during Pollination

**DOI:** 10.3390/cells11030475

**Published:** 2022-01-29

**Authors:** Bartosz J. Płachno, Małgorzata Kapusta, Piotr Stolarczyk, Anna Bogucka-Kocka

**Affiliations:** 1 Department of Plant Cytology and Embryology, Institute of Botany, Faculty of Biology, Jagiellonian University in Kraków, 9 Gronostajowa St., 30-387 Kraków, Poland; 2 Department of Plant Cytology and Embryology, Faculty of Biology, University of Gdańsk, 59 Wita Stwosza St., 80-308 Gdańsk, Poland; malgorzata.kapusta@ug.edu.pl; 3 Department of Botany, Physiology and Plant Protection, Faculty of Biotechnology and Horticulture, University of Agriculture in Kraków, 29 Listopada 54 Ave., 31-425 Kraków, Poland; piotr.stolarczyk@urk.edu.pl; 4 Chair and Department of Biology and Genetics, Medical University of Lublin, 20-093 Lublin, Poland; anna.kocka@umlub.pl

**Keywords:** carnivorous plants, female gametophyte, Lentibulariaceae, pectic homogalacturonan, xyloglucan

## Abstract

*Utricularia nelumbifolia* is a large carnivorous plant that is endemic to Brazil. It forms an extra-ovular female gametophyte, which surpasses the entire micropylar canal and extends beyond the limit of the integument and invades the placenta tissues. Due to the atypical behavior of the female gametophyte, it is interesting to determine the interaction between the gametophyte and sporophytic tissue. Therefore, the aim of this study was to evaluate the role of the placenta, the ovular tissues, the hypertrophied central cell and the integument in guiding the pollen tube in *Utricularia nelumbifolia* Gardner by studying the distribution of homogalacturonans and hemicelluloses. It was also determined whether the distribution of the homogalacturonans (HG) and hemicelluloses in *Utricularia* are dependent on pollination. The antibodies directed against the wall components (anti-pectin: JIM5, JIM7, LM19, LM20 and the anti-hemicelluloses: LM25, LM11, LM15, LM20, LM21) were used. Because both low- and high-esterified HG and xyloglucan were observed in the placenta, ovule (integument, chalaza) and female gametophyte of both pollinated and unpollinated flowers, the occurrence of these cell-wall components was not dependent on pollination. After fertilization, low methyl-esterified HGs were still observed in the cell walls of somatic cells and female gametophyte. However, in the case of high-esterified HG, the signal was weak and occurred only in the cell walls of the somatic cells. Because xyloglucans were observed in the cell walls of the synergids and egg cells, this suggests that they play a role in sexual reproduction. *Utricularia nelumbifolia* with an extra ovule-female gametophyte is presented as an attractive model for studying the male-female dialogue in plants.

## 1. Introduction

Together with hemicelluloses, pectins are an important polysaccharide component of the cell wall and have an effect on its properties (cell wall extensibility and porosity). Pectins play a role in many important processes, e.g., growth, morphogenesis, organogenesis, cell–cell adhesion, defense, leaf abscission and fruit and seed development, as well as ion binding. They can be divided into five main groups: homogalacturonan (HG), xylogalacturonan (XGA), apiogalacturonan (AP) and rhamnogalacturonan I and II [1,2,3]. The methyl-esterification and demethyl-esterification of homogalacturonans are key in plant development and the plant lifecycle [3]. In seed plants, there is a specific dialogue between the sporophyte and gametophytes and between the male gametophyte and female gametophyte e.g., [4,5,6,7,8]. The sporophytic tissues provide the specific ions and molecules that are necessary for the nutrition, attraction and guidance of the male gametophyte (pollen tube), including the calcium ions, arabinogalactan proteins (AGP) and homogalacturonans [9,10,11,12,13,14,15,16]. It is well known that homogalacturonans participate in the interaction between the pollen tube (the male gametophyte) and the pistil transmitting tract in various *Angiospermae* species [10,11,12,17,18,19,20]. However, the role of the homogalacturonan in the final stage of the progamic phase and during fertilization has only been studied in a few genera and species [21,22,23,24]. Thus, comparative studies of a phylogenetically broad range of taxa are required in order to understand the diversity of the homogalacturonans and their functions in different types of ovules and gametophytes. Generally, there are pollination- and fertilization-induced changes in the occurrence of both highly and low methyl-esterified homogalacturonans in the pistil transmitting tract, the micropyle of the ovule and also in the apoplast of the embryo sac cells. For example, Niedojadło et al. [22] showed that fertilization in *Hyacinthus orientalis* L. was followed by a change in the pectic composition in the filiform apparatus, the egg apparatus apoplast and also in the ‘‘micropylar’’ region of the central cell. The situation is more complicated in apomicts; Gawecki et al. [24] did not observe an abundant occurrence of highly methyl-esterified HG, which is labeled by the JIM7 antibody in either the micropyle or the synergids of the apomictic *Taraxacum*. Kościńska-Pająk [25] did not record HG (recognized by the JIM7 and JIM5 antibodies) in the micropylar part of the embryo sac, nor in the cytoplasm of the synergids in the apomict *Chondrilla juncea* L.

Even less is known about the participation of the hemicelluloses in plant sexual reproduction [21,24,26,27]. Mendes et al. [21] detected hemicelluloses in the denser matrix of the filiform apparatus in *Pitcairnia encholirioides* L.B.Sm. In the apomictic *Taraxacum*, Gawecki et al. [24] observed xyloglucan in the integumentary epidermis near the apex of the embryo sac and in the cytoplasm and extracellular matrix of the transmitting tissue cells, as well as heteromannan in the walls of the transmitting tissue cells and in some of the integument cells near the embryo sac and the micropylar canal. Wilson et al. [26] and Palmer et al. [27] recorded hemicelluloses in the cell walls of the endosperm. Therefore, the distribution and role of the hemicelluloses in the sexual plant organs (ovules, female gametophytes) are still unclear and require more research.

**Figure 1:**
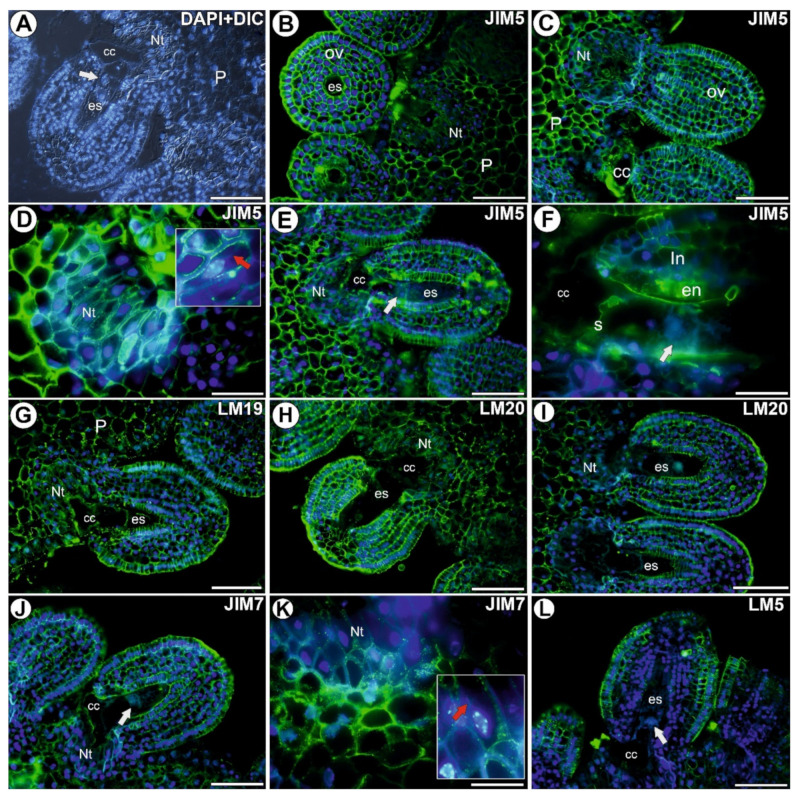
Histology and detection of the homogalacturonan in an ovule of *Utricularia nelumbifolia* before pollination (Antibodies–green fluorescence). (**A**) Section through the ovule and placenta that was stained with DAPI blue fluorescence and with differential interference contrast (DIC) optics. Note the presence of a mature embryo sac (es) with an egg cell (white arrow), hypertrophied part of the central cell (cc), placental nutritive tissue (Nt), placenta (P), bar 50 µm. (**B**,**C**) Pectic epitope (labeled with JIM5) in the ovule (ov) and placenta (P); mature embryo sac (es), hypertrophied part of the central cell (cc), bars 50 µm. (**D**) Pectic epitope (labeled with JIM5) in the placental nutritive tissue (Nt), the framed part shows the nuclei with the chromatubule (red arrow), bar 20 µm. (**E**,**F**) Pectic epitope (labeled with JIM5) in the embryo sac (es); hypertrophied part of the central cell (cc), synergid (s), egg cell (white arrow), endothelium (en), integument (In), placental nutritive tissue (Nt), bar 50 µm and bar 20 µm. (**G**) Pectic epitope (labeled with LM19)detected; placenta (P); mature embryo sac (es), hypertrophied part of the central cell (cc), bar 50 µm. (**H**,**I**) Pectic epitope (labeled with LM20) detected; mature embryo sac (es), hypertrophied part of the central cell (cc), placental nutritive tissue (Nt), bar 50 µm. (**J**) Pectic epitope (labeled with JIM7) detected; egg cell (white arrow), hypertrophied part of the central cell (cc), placental nutritive tissue (Nt), bar 50 µm. (**K**) Pectic epitope (labeled with JIM7) in the placental nutritive tissue (Nt), the framed part shows the nuclei with the chromatubule (red arrow), bar 20 µm. (**L**) Pectic polysaccharides (1-4)-β-D-galactan (labeled with LM5) detected; embryo sac (es), egg cell (white arrow), hypertrophied part of the central cell (cc), bar 50 µm.

*Utricularia* genus is remarkable in many ways [28]: for its carnivory, for its unique vegetative body plan [29,30,31] and for its reduction in non-genic nuclear DNA [32], but also for its embryological characters [33,34,35]. In the *Utricularia nelumbifolia* that was examined here, the central cell was hypertrophied at the micropylar pole and invaded the placenta (Figure 1A), and therefore the embryo sac was partially extra-ovular. In our previous work, we used this species to determine the distribution of the AGP and also to determine whether the production of the AGP is dependent on pollination [36]. Most of the detailed research on the role of the cell-wall components in the reproduction of flowering plants is based on several model species with a similarly developing embryo sac. Here, we propose a species with an extra-ovular female gametophyte as an attractive model for studying the male–female dialogue in plants.

Therefore, the aim of this study was to evaluate the role of the placenta, the ovular tissues and the hypertrophied central cell, as well as the integument in pollen tube guidance in *U. nelumbifolia* Gardner, by studying the production of homogalacturonan and hemicelluloses. It was also determined whether the distribution of homogalacturonan and hemicelluloses in *U. nelumbifolia* is dependent on pollination.

## 2. Materials and Methods

The *U.* *nelumbifolia* Gardner flowers were obtained from the living collections of the Jagiellonian University Botanical Garden (Kraków, Poland) in June 2018 and 2019. The studies were conducted on the flowers during anthesis and in pollinated flowers that were collected six, seven and nine hours after pollination. The plant material was fixed overnight in 8% (*w*/*v*) paraformaldehyde (PFA) with 0.25% (*v*/*v*) glutaraldehyde (GA) in a PIPES buffer at 4° C. It was then embedded in Steedman’s wax or LR White Resin (Polysciences Europe GmbH, Hirschberg an der Bergstrasse, Germany), and sectioned. The rehydrated sections were blocked with 1% BSA in a PBS buffer and incubated with the primary antibodies against anti-pectin: JIM5, JIM7, LM19, LM20 [37,38] www.kerafast.com, (accessed on 1 December 2021); anti-hemicelluloses: LM25, LM11, LM15 and mannans: LM21, LM22 [39,40] www.kerafast.com (accessed on 1 December 2021)] overnight at 4 °C. All of the primary antibodies were used in a 1:20 dilution and were purchased from Plant Probes, UK and www.kerafast.com (Kerafast, Inc., Boston, MA, USA), and the secondary antibody goat anti-rat conjugated with FITC was purchased from Abcam (Abcam plc, Cambridge, UK). The chromatin in the nuclei was stained with 7 µg/mL DAPI, and the samples were then cover-slipped using a Mowiol medium. They were viewed using a Nikon Eclipse E800 microscope (Tokyo, Japan) equipped with a B-2A filter, a GFP custom filter and a UV-2A filter. At least two different replications were performed, and the developmental stage of the analyzed flowers and about five to ten sections were analyzed from each structure for each antibody that was used. Negative controls were created by omitting the primary antibody step, which caused no fluorescence signal in the control frames for any of the stained slides (Supplementary Material Figrue S1). Semi-thin sections (0.9–1.0 μm thick) were prepared for light microscopy and stained for general histology using aqueous methylene blue/azure II (MB/AII) for 1–2 min as described in Płachno et al. [30].

## 3. Results

Heteromannan, which is detected by LM21 and LM22, was not observed in the examined material.

### 3.1. Homogalacturonan in the Placenta and Ovules before Pollination

A signal from the low methyl-esterified HG was observed in the cell walls of both the somatic cells and in the female gametophyte (Figure 1B–G). A strong and homogeneous signal of the fluorescence that is detected by JIM5 was observed in the cell walls of the integument and the placenta cells (Figure 1B,C). In the placental nutritive tissue cells, this epitope occurred in the part of the cell wall that borders the plasmalemma (Figure 1D). It was also detected in the embryo sac (Figure 1E) in the cell walls of the central cell and in the filiform apparatus (Figure 1F). The signal of the fluorescence detected by LM19 was observed in the cell walls of the integument and placenta cells and in the wall of the central cell (Figure 1G). In the nutritive tissue cells, the signal of the fluorescence detected by LM19 was observed as numerous small clusters (Figure 1G).

The signal from the highly esterified HG (detected by LM20), which occurred as numerous small dots, was observed in the walls of the somatic cells of both the integument and placenta (Figure 1H,I). The signal from the highly esterified HG (detected by JIM7) was observed in the walls of the cells of both the integument (intense signal in the endothelium) and the placenta, as well as in the central cell (Figure 1J,K). In the nutritive tissue cells, the nuclei had chromatubules (Figure 1K). A weak signal (a few small clusters) from this HG was recorded in the filiform apparatus of the synergids (Figure 1J). The signal from the HG by LM20 was observed in the cell walls of ovule cells when the mature female gametophyte was absent. In this case, this epitope occurred in the cell walls of the nucellus epidermis.

The signal from the pectic polysaccharides (1-4)-β-D-galactan (detected by LM5) was mainly found in the cell walls of the integument cells (Figure 1L). In the endothelium cells, this epitope occurred as clusters in the protoplasts.

### 3.2. Homogalacturonan in the Placenta and Ovules after Pollination and during Fertilization

**Figure 2:**
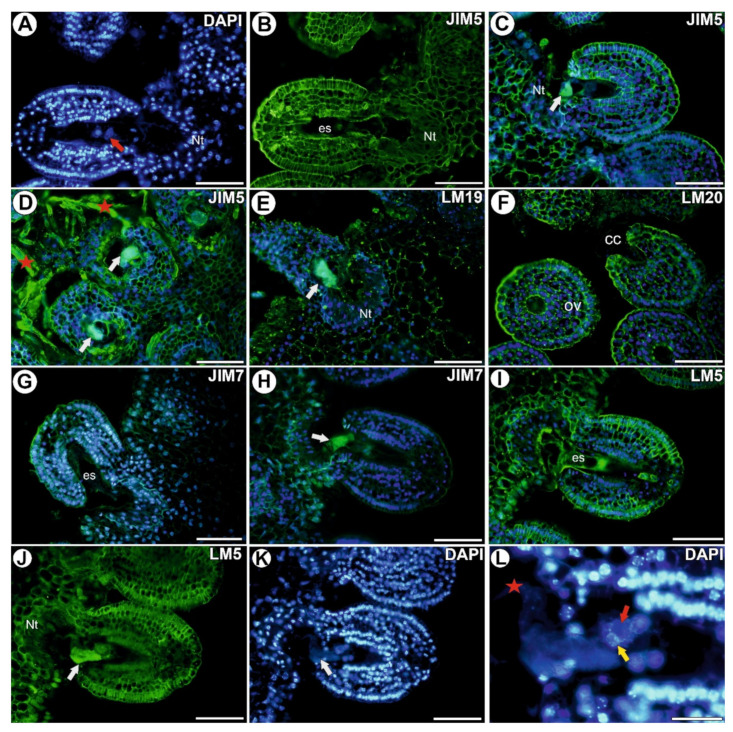
Homogalacturonan detected in an ovule of *Utricularia nelumbifolia* after pollination and after fertilization (Antibodies–green fluorescence). (**A**,**B**) Pectic epitope (labeled with JIM5) detected after pollination but before fertilization; mature embryo sac (es), egg cell (red arrow), placental nutritive tissue (Nt), bars 50 µm. (**C**,**D**) Pectic epitope (labeled with JIM5) detected after fertilization; synergid after it had been penetrated by the pollen tube (white arrow), pollen tube (star), placental nutritive tissue (Nt), bars 50 µm. (**E**) Pectic epitope (labeled with LM19) detected after fertilization; synergid after it had been penetrated by the pollen tube (white arrow), placental nutritive tissue (Nt), bar 50 µm. (**F**) Pectic epitope (labeled with LM20) after pollination but before fertilization; ovule (ov), hypertrophied part of the central cell (cc), bars 50 µm. (**G**) Pectic epitope (labeled with JIM7) after pollination but before fertilization; mature embryo sac (es), bar 50 µm. (**H**) Pectic epitope (labeled with JIM7) after fertilization; synergid after it had been penetrated by the pollen tube (white arrow), bar 50 µm. (**I**)) Pectic epitope (labeled with LM5) detected after pollination but before fertilization; mature embryo sac (es), bar 50 µm. (**J**,**K**) Pectic epitope (labeled with LM5) detected during fertilization; synergid after it had been penetrated by the pollen tube (white arrow), placental nutritive tissue (Nt), bar 50 µm. (**L**) Fertilization of the egg cell; note the nucleus of the sperm cell (yellow arrow) and egg cell nucleus (red arrow), pollen tube (red star), bar 20 µm.

After pollination, there were both fertilized and unfertilized ovules. In the ovules that had not been penetrated by the pollen tubes, low methyl-esterified HG were observed in the cell walls of both the somatic cells and in the female gametophyte (Figure 2A,B). In the fertilized ovules (after the pollen tube had penetrated the synergid),low methyl-esterified HG (detected by JIM5) were still observed in both the cell walls of somatic cells and in the cell walls of female gametophyte (Figure 2C,D). The accumulation of these HG occurred in the synergid that had been penetrated by the pollen tube (Figure 2C,D). These HG also occurred in the cell walls of the pollen tubes (Figure 2D). In the fertilized ovules, the signal of the fluorescence detected by LM19 was observed in the cell walls of the integument and in the cell walls of placenta cells (Figure 2E).

After pollination, the signal from the highly esterified HG (detected by LM20) was observed in the cell walls of both the somatic cells and in the cell walls of the female gametophyte (Figure 2F). In the case of the highly esterified HG (detected by JIM7), the signal was weak and occurred in the cell walls of the ovule cells (Figure 2G). After fertilization, the signal of this HG occurred in the outer cell walls of the epidermal cells at the chalazal pole of the ovule (Figure 2H).

After pollination, the signal from the pectic polysaccharides (1-4)-β-D-galactan (detected by LM5) was detected in the cell walls of both the somatic cells and in the central cell of the female gametophyte (Figure 2I), however, in the fertilized ovules (during fusion egg cell nucleus with sperm nucleus), it occurred only in the cell walls of the somatic cells (Figure 2J–L).

### 3.3. Hemicelluloses in the Placenta and Ovules before Pollination

**Figure 3:**
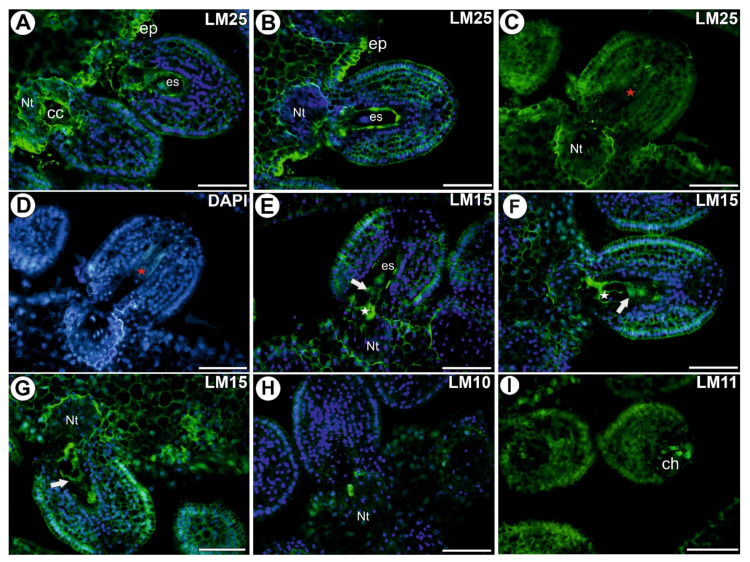
Xyloglucan detected in an ovule of *Utricularia nelumbifolia* before pollination (Antibodies–green fluorescence). (**A**,**B**) Xyloglucan (labeled with LM25) detected; note the intense signal in the epidermis of the placenta (ep); embryo sac (es), hypertrophied part of the central cell (cc), placental nutritive tissue (Nt), bars 50 µm. (**C**,**D**) Xyloglucan (labeled with LM25) detected, ovule without a female gametophyte; note the persistent epidermis of the nucellus (red star); placental nutritive tissue (Nt), bars 50 µm. (**E**–**G**) Xyloglucan (labeled with LM15) detection; note the intense signal in the synergids (white star), egg cell (white arrow); embryo sac (es), placental nutritive tissue (Nt), bars 50 µm. (**H**) Xylan (labeled with LM10) detected; placental nutritive tissue (Nt), bar 50 µm. (**I**) Xylan/arabinoxylan (labeled with LM11) detected; note the positive signal at chalaza (ch), bar 50 µm.

A signal from xyloglucan (labeled with LM25) was observed in the cell walls of both the somatic cells and the female gametophyte (Figure 3A–D). This xyloglucan occurred in the cell walls of the egg apparatus and in the central cell (Figure 3A). The signal was especially intense in the cell walls of the epidermal cells of the placenta (Figure 3A–D). In the ovule in which the female gametophyte development had stopped (Figure 3C,D), xyloglucan (detected by LM25) was present in the cell walls of ovule cells. The signal from xyloglucan (detected by LM15) was observed in the cell walls of the epidermal cell of the ovule, however, a very intense signal occurred in the cell walls of the synergids (also in the filiform apparatus), egg cell, and nucellus remained (Figure 3E–G). The signal from xylan (detected by LM10) was only observed in the cell walls of a few cells of the placenta or integument cells near the micropylar part of the female gametophyte (Figure 3H). The signal from xylan/arabinoxylan (detected by LM11) was observed in the cell walls of a few cells in the placenta near the micropylar part of the female gametophyte and in the cell walls of a few cells of the chalaza (Figure 3I).

### 3.4. Hemicelluloses in the Placenta and Ovules after Pollination and during Fertilization

**Figure 4:**
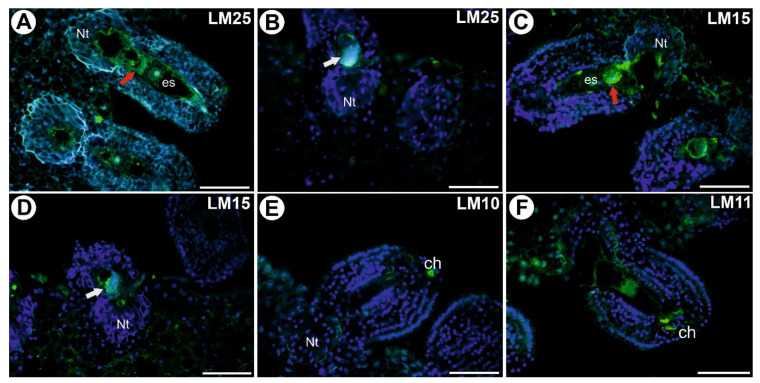
Xyloglucan detected in an ovule of *Utricularia nelumbifolia* after pollination and after fertilization (Antibodies–green fluorescence). (**A**) Xyloglucan (labeled with LM25) detected after pollination but before fertilization; embryo sac (es), egg cell (red arrow), placental nutritive tissue (Nt), bar 50 µm. (**B**) Xyloglucan (labeled with LM25) detected after fertilization; synergid after it had been penetrated by the pollen tube (white arrow), placental nutritive tissue (Nt), bar 50 µm. (**C**) Xyloglucan (labeled with LM15) detected after pollination but before fertilization; embryo sac (es), egg cell (red arrow), placental nutritive tissue (Nt), bar 50 µm. (**D**) Xyloglucan (labeled with LM15) detected after fertilization; synergid after it had been penetrated by the pollen tube (white arrow), placental nutritive tissue (Nt), bar 50 µm. (**E**) Xylan (labeled with LM10) detected after pollination but before fertilization; placental nutritive tissue (Nt); note the positive signal at the chalaza (ch), bar 50 µm. (**F**) Xylan/arabinoxylan (labeled with LM11) detected after pollination but before fertilization; note the positive signal at the chalaza (ch), bar 50 µm.

After the pollination, a signal from xyloglucan (detected by LM25) was observed in the cell walls of the placenta cells and in the female gametophyte (in the cells walls of the egg apparatus and central cell) (Figure 4A). In the fertilized ovule, this xyloglucan was observed in the cell walls of zygote (Figure 4B). After the pollination, a signal from xy-loglucan (detected by LM15) was observed in the cell walls of the egg cell and syner-gids (Figure 4C). In the fertilized ovules, the signal of LM15 was still observed near the synergid that had been penetrated by the pollen tube (Figure 4D). This xyloglucan was observed in the cell walls of pollen tubes (Figure 4D). After pollination, the signal from xylan (detected by LM10) was observed in the cell walls of a single cell at the chalaza (Figure 4E). The signal from xylan/arabinoxylan (detected by LM11, Figure 4F) was sim-ilar to the one in the unpollinated flowers.

## 4. Discussion

Niedojadło et al. [22] showed that in mature ovules of *Hyacinthus orientalis* L., the methyl-esterified HG that were detected by JIM7 were predominant. In *Quercus suber* L., Lopes et al. [23] observed a uniform distribution of the HG detected by JIM7 in all of the sporophytic cells of the ovule. In *Taraxacum*, in an ovule that contained a mature female gametophyte, the cell walls of the somatic cells were rich in low methyl-esterified HG, however, highly methyl-esterified HG also occurred [24,41]. Here, we showed that the somatic cells of the placenta and mature ovule contained extremely low methyl-esterified HG (intense signal of JIM5). Interestingly, Pérez-Pastrana et al. [42] observed that in *Capsicum chinense* Jacq. at early stages of ovule development, the pectin was highly methyl-esterified, however, the methyl-esterification decreased gradually throughout the process. Thus, in the mature ovule of this species, low methyl-esterified HG dominated. Unfortunately, although we did not study the changes in the HG during ovule development, it seems that the occurrence of low methyl-esterified HG and highly methyl-esterified HG in the mature ovules is species or genus specific. Moreover, there have been various results in the case of the occurrence of HG in the female gametophyte, particularly in the filiform apparatus. For example, Mendes et al. [21] studied the development of the filiform apparatus in the synergids of *Pitcairnia encholirioides* L.B.Sm. These authors showed that highly methyl-esterified HG were present in the filiform apparatus in contrast to low methyl-esterified HG. They proposed that the absence of the de-esterified HG in the filiform apparatus might indicate an ephemeral condition of this structure. HGs with a high degree of esterification (JIM7) and a low degree of esterification (JIM5) were observed in the filiform apparatus of *Hyacinthus orientalis* by Niedojadło et al. [22]. We also detected low methyl-esterified HG in the filiform apparatus, but the signal of the highly methyl-esterified HG was weak. However, it should be stressed that the *Utricularia* species develop a very specific filiform apparatus that is quite different than other more typical plants [34]. We observed both low and highly methyl-esterified HG in the cell wall of the central cell. Costa et al. [43] did not detect any low methyl-esterified HG in the cell wall of the central cell in *Trithuria submersa,* but did detect highly methyl-esterified HG there. Niedojadło et al. [22] observed both types of these HGs in the cell wall of the central cell in *Hyacinthus orientalis*.

We recorded both low and highly methyl-esterified HG in the cell walls of the nutritive tissue cells. In *Utricularia nelumbifolia*, this tissue is unusual because the nuclei of its cells have chromatubule-projections that contain chromatin and proteinaceous tubule-like inclusions [44], which we also observed in the examined material.

**Figure 5:**
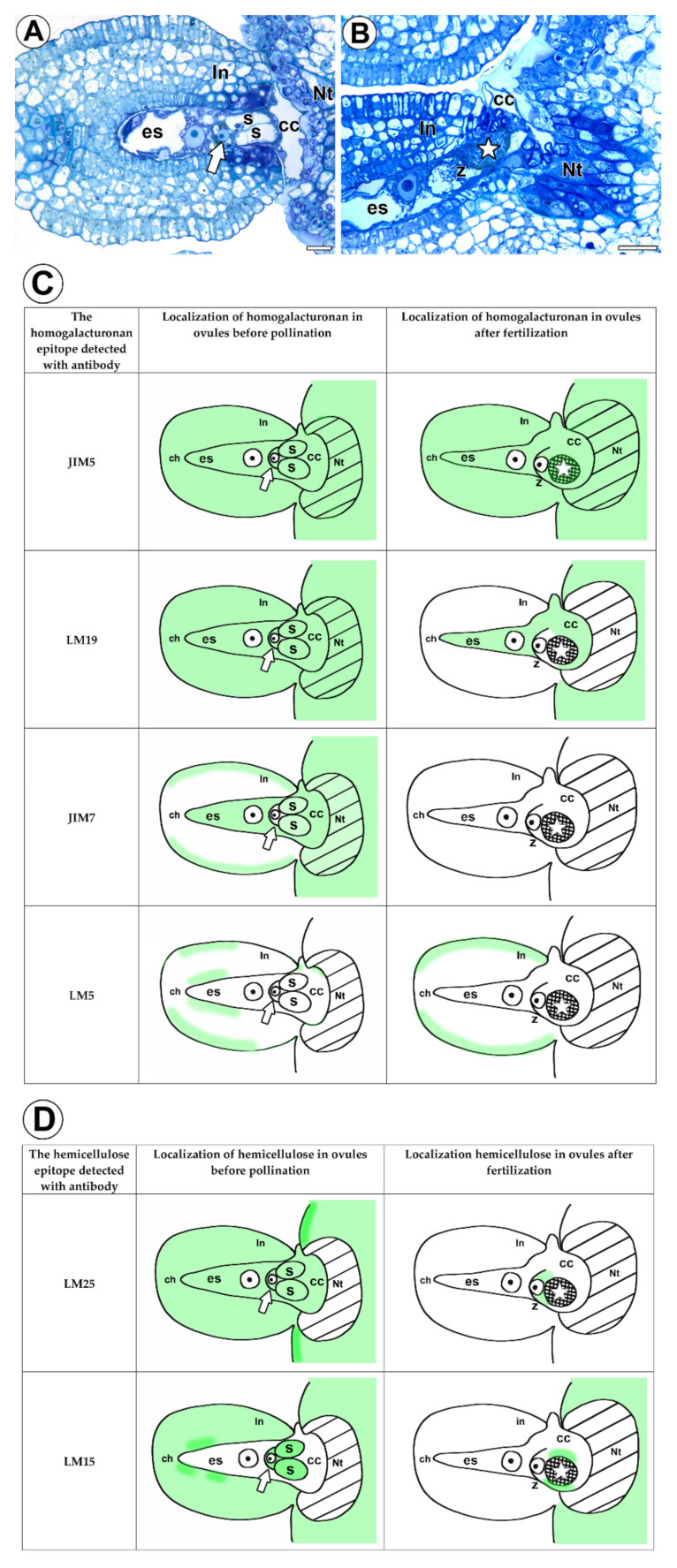
Histology of an ovule of *Utricularia nelumbifolia* before pollination and after fertilization. Schematic localization of the homogalacturonan and hemicelluloses in the female reproductive structures and tissues of *Utricularia nelumbifolia* before pollination and after fertilization. Dark color represents intense or average labelling. (**A**) Section through the ovule and placenta before pollination, egg cell (arrow), hypertrophied part of the central cell (cc), placental nutritive tissue (Nt), integument (in), chalaza (ch), bar 10 µm. (**B**) Section through the ovule and placenta after fertilization, egg cell (arrow), hypertrophied part of the central cell (cc), placental nutritive tissue (Nt), integument (in), chalaza (ch), zygote (z), synergid after it had been penetrated by the pollen tube (star), bar 20 µm. (**C**) Schematic localization of the homogalacturonan in the female reproductive structures and tissues of *Utricularia nelumbifolia* before pollination and after fertilization, egg cell (arrow), hypertrophied part of the central cell (cc), placental nutritive tissue (Nt), integument (in), chalaza (ch), zygote (z), synergid after it had been penetrated by the pollen tube (star). (**D**) Schematic localization of the hemicelluloses in the female reproductive structures and tissues of *Utricularia nelumbifolia* before pollination and after fertilization, egg cell (arrow), hypertrophied part of the central cell (cc), placental nutritive tissue (Nt), integument (in), chalaza (ch), zygote (z), synergid after it had been penetrated by the pollen tube (star).

We showed that after pollination and fertilization, low methyl-esterified HGs were still observed in the somatic cells and female gametophyte (Figure 5A–C). However, in the case of the highly esterified HGs (detected by JIM7), the signal was weak and only occurred in the somatic cells. Moreover, after fertilization the pectic polysaccharides (1-4)-β-D-galactan only occurred in the somatic cells. In *Utricularia nelumbifolia*, these changes are also correlated with the AGP distribution. In a previous work [36], we showed that the penetration of the embryo sac by the pollen tube and the process of fertilization changed the pattern of the AGP (labeled by JIM13 and JIM8) distribution in this species dramatically. Niedojadło et al. [21] showed that in *Hyacinthus orientalis*, pollination induced the rearrangement of the HG in the egg apparatus, and also that fertilization led to further changes in their distribution. It is considered that the methyl-esterification status of HG can have consequences for cell wall texture and mechanical properties [3]. The weakly methyl-esterified HG form complexes with divalent calcium ions. These “egg box” complexes play a significant role in the structural rigidity of the cell wall and in mediating cell-to-cell adhesion e.g., [45,46,47]. It seems that “egg box” complexes are critical in plant sexual reproduction, e.g., in mediating the interaction between pollen tube and sporophytic tissues [9,10,11,19]. Thus a rearrangement of the HG in the cell walls of the *Utricularia* embryo sac after pollination may represent preparation to receive a pollen tube, creating an optimal environment for directed growth of the pollen tube and for final entry of the male gametophyte into the embryo sac. However, changes in the structure of cell walls probably also have an effect on the flow of nutrients and signaling molecules between the sporophytic tissues and an embryo sac. Both these results of a rearrangement of the HG were earlier suggested by Niedojadło et al. [22] in regard to *Hyacinthus orientalis*. However, in case *Utricularia* this needs further studies.

HG also play a significant role during plant seed development. The best-studied myxospermic species is *Arabidopsis thaliana* (L.) Heynh. [48,49], in which large amounts of pectinaceous mucilage are deposited into a specific domain of the cell wall [50]. In this species, HG mucilage consists of a water-soluble outer layer and an adherent inner layer. The inner layer can be separated into domains of HG with different levels of methyl esterification [51]. According to Francoz et al. [52], partially demethyl-esterified pectin patterns act as a platform allowing positioning of PEROXIDASE36 in a remote primary cell wall domain during early development of *Arabidopsis* mucilage cells. These authors showed that changes in the methyl-esterification of HG are required for the loosening of this domain during later development, and finally for successful seed imbibition and germination. Therefore, the seed mucilage (including HG) may not only influence seed-dispersal but may also enhance soil-water retention, hydraulic conductivity and stability, thus helping in seed germination [53].

In both angiosperms and gymnosperms, HG were observed in the pollen tubes [54], however, there are differences between these two groups. In angiosperms, low methyl-esterified HG is a main component of the pollen tube wall, but in some gymnosperm species the highly methyl-esterified HG dominates; see [55] and references therein. Here, we observed minimally methyl-esterified HG and also xyloglucan in the pollen tubes of *Utricularia nelumbifolia*. Xyloglucan (detected by LM15) was also observed in the pollen tubes of other angiosperms, e.g., *Nicotiana alata* [56].

As mentioned earlier, hemicelluloses have rarely been studied in flowering plant embryology (distribution in the female gametophyte before and after pollination). Mendes et al. [21] used the LM15 and LM21 antibodies to label the hemicelluloses in the female gametophytes of *Pitcairnia encholirioides*. They observed them in the filiform apparatus. We also observed xyloglucan in the synergids. However, in *Utricularia nelumbifolia*, xyloglucan also occurred in the egg cell and central cell (Figure 5A–D). The presence of hemicelluloses in the synergids in a distant, unrelated taxa indicates their universal role in the filiform apparatus, showing that they play a role in the sexual reproduction of angiosperms. In contrast to Mendes et al. [21], we did not detect mannan in either the female gametophyte or the somatic cells.

The signal of LM25 was especially intense in the epidermal cells of the placenta. In a previous work [36], we showed that AGPs occurred in the epidermis of the placenta of *Utricularia nelumbifolia*. The occurrence of hemicelluloses in these cells supports our hypothesis that the placenta epidermis forms a transmission track for the pollen tubes. In *Taraxacum*, Gawecki et al. [24] recorded hemicelluloses in the somatic cells, including transmitting tissue cells.

We found highly esterified HG and xyloglucan in the ovules in which the development of the female gametophyte had stopped. It seems that the production or occurrence of some highly esterified HG and xyloglucan in the ovular tissues (nucellus, integument) was not dependent on the presence of a mature embryo sac. However, this needs future study, especially because these cell-wall components could be produced in the early stages of ovule development and simply persist in this unfunctional ovule in mature flowers. 

To the best of our knowledge, this study is the first to show the occurrence of homogalacturonans and hemicelluloses in the generative structures in Lentibulariaceae. Both low and highly esterified HGs and xyloglucan were observed in the placenta, ovule (integument, chalaza) and female gametophyte of both pollinated and unpollinated flowers, thus, it can be concluded that the occurrence of these cell-wall components is not dependent on pollination. Xyloglucans were observed in the synergids and egg cell, which suggests that they play a role in sexual reproduction. Highly esterified HG and xyloglucan occurred in the ovules in which the development of the female gametophyte had stopped, thus, it seems that the production of these wall components in the ovular tissues (nucellus, integument) was not dependent on the presence of a mature embryo sac. However, this needs further study.

## Supplementary Materials

The following are available online at https://www.mdpi.com/article/10.3390/cells11030475/s1, Figure S1: Control reactions of the immunolabeling of the cell wall components.
