# Supplementary material for: Spatiotemporal Distribution of Homogalacturonans and Hemicelluloses in the Placentas, Ovules and Female Gametophytes of Utricularia nelumbifolia during Pollination
Source: Cells. 2022 Jan 29;11(3):475. doi: 10.3390/cells11030475 (PMC8834615; doi:10.3390/cells11030475)
Supplement: Supplementary file 1 [file cells-11-00475-s001.zip › cells-1542579-supplementary.pdf]

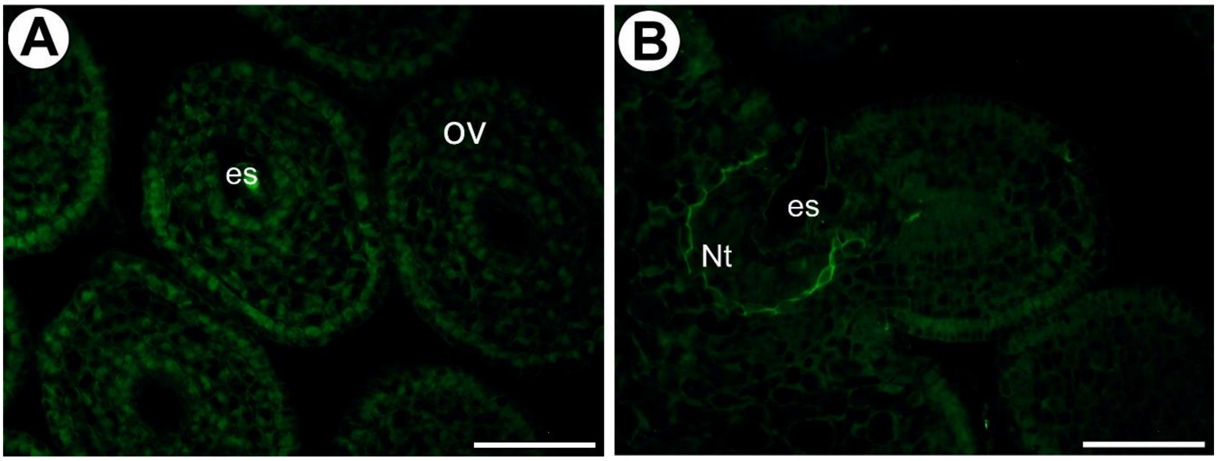

Figure S1. Control reactions of the immunolabeling of the cell wall components. (**A,B**) ovule (ov), embryo sac (es), placental nutritive tissue (Nt), bars 50 μm.
